# Enhanced efficiency of bifacial perovskite solar cells using computational study

**DOI:** 10.1038/s41598-024-62487-0

**Published:** 2024-06-06

**Authors:** Mohammad Istiaque Hossain, Puvaneswaran Chelvanathan, Amith Khandakar, Kevin Thomas, Ahasanur Rahman, Said Mansour

**Affiliations:** 1 HBKU Core Labs, Hamad Bin Khalifa University (HBKU), Qatar Foundation, Doha, Qatar; 2 Solar Energy Research Institute (SERI), National University of Malaysia (UKM), Bangi, Malaysia; 3 Department of Electrical Engineering, College of Engineering, Qatar University, Doha, Qatar

**Keywords:** Perovskite solar cell, Bifacial structure, SCAPS, Computational study, Operating temperature, Energy science and technology, Materials science, Nanoscience and technology

## Abstract

The most rapidly expanding type of solar cells are the Perovskite Solar Cells (PSCs), because of its high device performance, ease of synthesis, high open-circuit voltage, and affordability. Despite these advantages, the development of perovskite-based solar cells continues to be impeded by the issues with perovskite stability and the utilization of the hazardous heavy element lead (Pb). The study emphasizes on the bifacial structure that maintains the conventional absorber layer and electron transport layer (ETL) in the optimized PSC structure. This study employs SCAPS software for device simulation to comprehensively analyze how various parameters affect the performance of solar cells. Additionally, doping concentration variation in both ETL and HTL are explored. The simulation reveals that changing device structure from monofacial to bifacial significantly influences PSC performance, demonstrating that optimizing individual layers effectively enhances overall solar cell performance. The optimized structure achieves impressive PSC performance metrics through parametric analysis, such as voltage (V_OC_) of 1.18 V, fill factor (FF) of 82.24%, current density (J_SC_) of 27.12 mA/cm^2^, power conversion efficiency (PCE) of 27.90% for an incident solar spectrum from the ETL side, and power conversion efficiency (PCE) of 19.86% for an incident solar spectrum from the HTL side, the calculated bifaciality factor (BF) for this structure is 71.18%.

## Introduction

**Figure 1:**
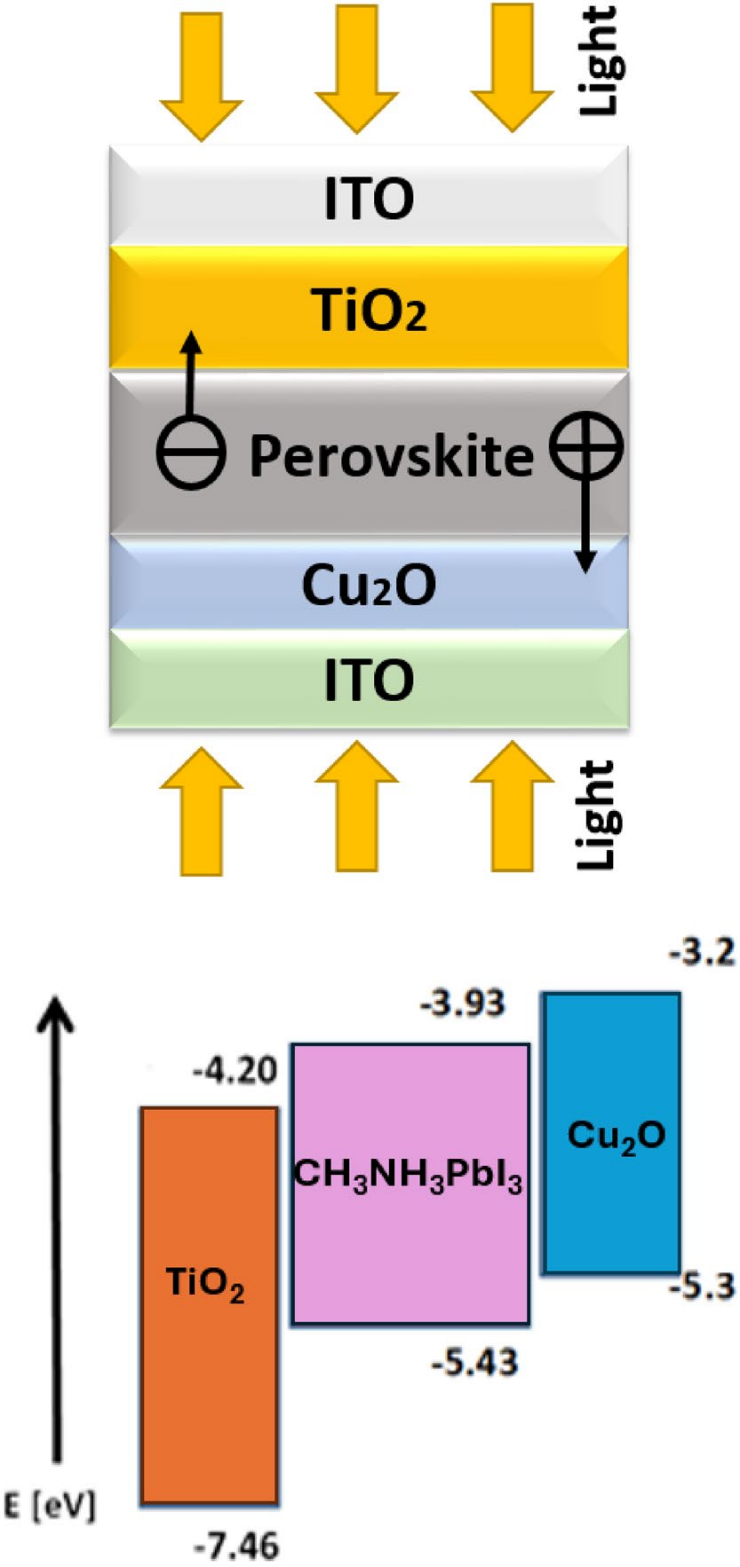
(**a**) ITO/TiO_2_/perovskite/Cu_2_O/ITO device structure, (**b**) band diagram of the device.

During the past several years, the rapid progress of Perovskite Solar Cells (PSCs) have drawn significant attention in solar cell research, displaying promising advancements in both cost-effectiveness and efficiency. Introduced by Lev Perovskite in 2009, the initial PSC exhibited a modest efficiency of 3.8%^1,2^. However, within a decade, PSC efficiency skyrocketed to a remarkable 23.7% for Methyl Ammonium Lead Halide (CH_3_NH_3_PbI_3_) perovskite-based cells^3^. Perovskite materials are suitable for photovoltaic effects because they have a long dispersion charge carrier length, low excitation binding energy, improved mobility of charge carriers, and high absorption coefficient^4^. But it's important to recognize that the specific lead-containing perovskite compounds, such MAPbI_3_, raise questions about stability and environmental effect, which might prevent them from being widely used in solar cell technology. Development of a bifacial structure is one way to address this issue, utilizing superior optoelectronic characteristics including a less exciton binding energy, better absorption coefficient, and higher carrier transport^5–7^. This bifacial structure corresponds to a group in the periodic table that is similar to its lead-containing counterpart, with a direct bandgap of 1.3 eV falling within the absorber range^8^.The fundamental layers of the typical PSC structure are contact electrodes, transparent glass, the hole transport layer (HTL), the absorber layer (Abs), and the electron transport Layer (ETL) (see Fig. 1). In addition to extracting and transporting photo generated electron carriers, the ETL inhibits charge recombination by acting as a hole-blocking layer^9^. Due to its advantageous bandgap alignment, excellent electron mobility, chemical stability, and compatibility with various deposition methods, titanium dioxide (TiO_2_) is commonly employed as an electron transport layer (ETL)^10^ whereas HTL serves as an electron-blocking layer, facilitating hole flow while impeding electron movement^11^. To achieve the maximum power conversion efficiency, both layers are essential and require immaculate layers with low bulk and interface defects. The quest for improved stability and performance in perovskite solar cells emphasizes the importance it is to switch from conventional or tandem structures to a bifacial structure, particularly for applications such as Vehicle Integrated Photovoltaics (VIPV) and Building Integrated Photovoltaics (BIPV)^2^. This paper advocates the development of stable and effective bifacial solar cells designed to be cost-effective, with potential scalability in manufacturing techniques, as depicted in Fig. 1. Figure 1b shows the device band structure for carrier injection.

While numerous publications globally, including those in Australia, have explored monofacial and bifacial tandem perovskite solar cells^12–18^, ongoing research actively addresses the pursuit of stability, simplified structures, and enhanced light management^19–21^. Notably, recent innovations like the bifacial tandem solar cell developed by the Energy Research Centre of the Netherlands (ECN)^22^ with a conversion efficiency of 30.2%, highlight the potential of bifacial structures in achieving superior performance. However, the complexity and cost associated with fabricating such structures present significant challenges, including lattice matching, current continuity, band structure alignment, and management of photo-generated carrier tunneling between junctions^23–33^. The study explores optimizing bifacial Perovskite Solar Cells (PSCs), offering new insights into their potential applications and performance enhancements. By using SCAPS software, the research conducts comprehensive simulations to analyze various parameters affecting PSC performance, providing cost-effective insights into device structures and material properties. The paper systematically optimizes individual layers within the PSC structure, such as the absorber layer, ETL, and HTL, to enhance overall solar cell efficiency. Investigating the effects of operating temperature on bifacial solar cell performance adds valuable insights for real-world applications, helping understand temperature's influence on key parameters. The study explores novel materials like copper oxide (Cu_2_O) as potential HTMs, suggesting alternatives to traditional materials and addressing environmental and stability concerns. The paper discusses practical implications, such as scalability of manufacturing techniques, and highlights the importance of experimental validation for novel materials and device structures, paving the way for future research in bifacial perovskite solar cells. All of them combined to see the performance of the perovskite solar panel can give us insight on the feasibility of this kind of solar panel.

Increasing the thickness of the absorber layer enhances light absorption by allowing more photons to be absorbed. However, thicker absorber layers can also increase carrier diffusion lengths, leading to longer transit times and potentially higher recombination rates if charge extraction pathways are not optimized. The design of the bifacial structure, including the arrangement of layers and interfaces, influences both light absorption and charge extraction efficiency. Design choices that enhance one aspect may inadvertently compromise the other, highlighting the need for comprehensive optimization strategies. Increasing light absorption tends to improve energy yield by capturing more sunlight. However, modifications to enhance charge extraction efficiency, such as additional layers or contacts, may impact the visual transparency or aesthetic appeal of the bifacial structure, particularly in applications like building-integrated photovoltaics (BIPV).Trade-offs between performance improvements and practical considerations must be carefully evaluated to ensure commercial viability.

Metal oxides often exhibit high electron mobility, allowing for efficient transport of electrons within the device^3,7^. This facilitates rapid charge carrier extraction, reducing recombination losses and improving overall device performance. The energy levels of metal oxides can be tailored through dopants or adjustments in composition, enabling alignment with the energy levels of adjacent layers in the solar cell. This alignment enhances charge carrier extraction and reduces energy loss at interfaces. Also, Metal oxides are generally chemically stable under operating conditions, resisting degradation from exposure to moisture, oxygen, and light. This stability contributes to the long-term reliability and durability of the solar cell. Some metal oxides, such as indium tin oxide (ITO) and fluorine-doped tin oxide (FTO), exhibit both high electrical conductivity and optical transparency. This combination allows them to serve as transparent conductive electrodes, enabling light to penetrate the active layer while efficiently collecting charge carriers. Metal oxides are abundant and cost-effective, making them attractive materials for large-scale production of solar cells. Their availability contributes to reducing the overall manufacturing cost of solar panels, thus improving the economic viability of solar energy technology. Metal oxides can serve multiple functions within a solar cell, acting as electron transport layers, hole blocking layers, or even as part of the active layer in certain types of photovoltaic devices. This versatility allows for the design of customized device architectures tailored to specific performance requirements.

This work investigates the application of bifacial Perovskite Solar Cells with Cu_2_O serving as the Hole Transport Layer (HTL) and TiO_2_ serving as the Electron Transport Layer (ETL) in order to overcome these difficulties. By analyzing different parameters for every PSC layer, the suggested numerical simulations, carried out with the 1-D solar cell capacitance simulator (SCAPS), direct the fabrication process to reduce costs and increase cell efficiency. To maximize the performance of each layer (ETL, Abs, and HTL), important parameters such as power conversion efficiency (PCE), fill factor (FF), short-circuit current (J_SC_), and open-circuit voltage (V_OC_) are analyzed. In search of the ideal setup, the study explores variables including type, thickness, defect density, doping concentration, and operating temperature. Additionally, the performance of PSC under varying incoming light concentration is explored in numerical analyses conducted under AM1.5G light conditions using the SCAPS simulator. In these simulations, the intensity of the incoming light is varied using the transmission option in SCAPS. This option allows for the simulation of different light conditions by adjusting the transmission coefficient.

## Methodology

Developed at ELIS, University of Gent, the one-dimensional solar cell simulation program SCAPS-1D was used in this work^14^. Curves derived from Poisson Eq. (1) and the continuity equations for electrons (2) and holes (3) under steady-state conditions are applied to compute the output of the simulated device structure.

1 $$\frac{d}{dx}\left(-\varepsilon \left(x\right)\frac{d\varphi }{dx}\right)=q\left[p\left(x\right)-n\left(x\right)+{N}_{D}^{+}\left(x\right)-{N}_{A}^{-}\left(x\right)+{p}_{t}\left(x\right)-{n}_{t}\left(x\right)\right]$$

2 $$\frac{{dp}_{n}}{dt}={G}_{p}-\frac{{p}_{n}-{p}_{n0}}{{\tau }_{p}}+{p}_{n}{\mu }_{p}\frac{d\xi }{dx}+{\mu }_{p}\xi \frac{{dp}_{n}}{dx}+{D}_{p}\frac{{d}^{2}{p}_{n}}{{dx}^{2}}$$

3 $$\frac{{dn}_{p}}{dt}={G}_{n}-\frac{{n}_{p}-{n}_{p0}}{{\tau }_{n}}+{n}_{p}{\mu }_{n}\frac{d\xi }{dx}+{\mu }_{n}\xi \frac{{dn}_{p}}{dx}+{D}_{n}\frac{{d}^{2}{n}_{p}}{{dx}^{2}}$$

The following definitions of various parameters and the symbols that correspond to them are used in this study: The generation rate is represented by G, and the lifetimes of electrons and holes are indicated by τn and τp, respectively. The symbols D and q represent the diffusion coefficient and electron charge, respectively. Ψ stands for the electrostatic potential, while μn and μp denote the mobilities of electrons and holes. Additionally, n(x) and p(x) represent the concentrations of free electrons and holes, while nt(x) and pt(x) indicate the concentrations of trapped electrons and holes. N − A(x) and N + D(x) correspond to the concentration of ionized acceptors and donors, ξ represents the electric field, and x signifies the direction along the thickness^15^. In this study, a two-stage PSC model device structure was created, consisting of an optimized device structure and a conventional device structure with light shining from the back. ITO/Cu_2_O/Perovskite/TiO_2_/ITO is the traditional device structure used in this work, which acts as a baseline for the investigation and PSC device structure optimization. The TiO_2_ layer was the primary focus of the optimization process, which also investigated other TiO_2_ parameters like defect density, thickness, and operating temperature. The back contact layers, Abs layer, and HTL were optimized concurrently, encompassing the investigation of various types and thicknesses. The optimized structural design was then examined in the presence of dark currents. The study also looked into how the absorber layer's defect density and doping concentration affected the PSC's efficiency. The simulation was carried out with 1000 W/m^2^ of AM 1.5G air mass illumination. In this study, the Power Conversion Efficiency (PCE) analysis of the PSC with different parameters served as the basis for optimizing each layer. The optimization process commenced with the ETL optimization, involving simulations with varying operating temperatures, thicknesses, and defect densities. Subsequently, the absorber layer was optimized, with thicknesses varying between 100 and 1k nm. Table 1 contains the parameters that are used for the different layers^16–20^. Using an optimized device structure after each PSC layer was optimized, dark current analysis was performed and examined, as illustrated in Fig. 3. The study also looked into how the absorber layer's defect density and doping concentration affected recombination rate and performance. We varied the absorber layer's doping concentration and defect density from 10^14^ to 10^19^ cm^−3^ and 10^14^ to 10^18^ cm^-3^, respectively.

**Table 1:** Parameters used in SCAPS.

Parameters	Layer used		
	Cu_2_O	Perovskite	TiO_2_
Bandgap (eV)	2.200	1.500	3.260
Electron affinity (eV)	3.200	3.900	4.000
Dielectric permittivity	9.400	10.000	10.000
CB (1/cm^3^)	8.000E+17	2.000E+18	2.800E+18
VB (1/cm^3^)	1.800E+19	1.000E+18	1.000E+18
Electron thermal velocity (cm/s)	1.000E+7	1.000E+7	1.000E+7
Hole thermal velocity (cm/s)	1.000E+7	1.000E+7	1.000E+2
Electron mobility (cm^2^/Vs)	2.000E+2	1.000E+2	1.000E+2
Hole mobility (cm^2^/Vs)	1.000E+2	1.000E+1	2.500E+1
Donor density ND (1/cm^3^)	0.000E+0	1.000E+9	1.100E+17
Acceptor density NA (1/cm^3^)	2.000E+14	1.000E+9	0.000E+0

The defect density behind the SCAPS (Solar Cell Capacitance Simulator) solar cell model refers to the concentration of imperfections or irregularities within the semiconductor material used in the device. These defects can include vacancies, interstitials, dislocations, and impurities, among others, which can impact the electronic properties and performance of the solar cell. Understanding and controlling defect density is crucial for optimizing the efficiency and reliability of solar cells. High defect densities can lead to increased recombination of charge carriers, reducing the overall efficiency of the device. Additionally, defects can introduce trap states within the bandgap of the semiconductor, which can hinder charge carrier transport and collection, further decreasing device performance. In the context of the SCAPS model, incorporating defect density data allows researchers and engineers to simulate and analyze the effects of defects on the electrical behavior of the solar cell. By adjusting parameters related to defect density in the simulation, such as trap state concentrations or defect energy levels, researchers can assess how different defect configurations impact device performance, including factors like open-circuit voltage, short-circuit current, and fill factor. Furthermore, understanding the relationship between defect density and device performance enables researchers to design strategies for defect mitigation and passivation. Techniques such as defect engineering, surface passivation, and material purification can help reduce defect densities and improve the efficiency and reliability of solar cells.

In summary, defect density behind the SCAPS solar cell model plays a critical role in understanding and optimizing the performance of photovoltaic devices. By incorporating defect density data into simulations and analyses, researchers can gain insights into the effects of defects on device behavior and develop strategies to mitigate their impact, ultimately enhancing the efficiency and reliability of solar cells.

For the fabrication part, we can acquire all necessary chemicals from Sigma Aldrich™ and utilize them without further processing. For the project at hand, utilize Indium-doped tin oxide (FTO) coated borosilicate glass substrates measuring 3 cm × 3 cm × 1.5 mm. Before deposition, systematically clean the substrates in an ultrasonic bath for 5 min each with acetone, isopropanol, and deionized water. Subsequently, dry the samples using nitrogen blow and proceed directly to deposition. Cu_2_O films can be deposited via DC magnetron sputtering at a power density of 1.5 W/cm^2^, utilizing a Cu target of 99.999% purity. Deposition temperatures can vary from 100 °C to 250 °C under oxygen and nitrogen flow, maintaining a background pressure of approximately 10 mTorr, with a base pressure around 10–5 Torr achieved by the turbo molecular pump. A typical film thickness of 100 nm is attainable, ensuring uniform structural and morphological characteristics across the entire sample surface with optimized optical and structural properties. The perovskite layer can be formed atop the Cu_2_O layer via thermal evaporation using PbI_2_ (Sigma-Aldrich) and CH_3_NH_3_I (Dyesol) source powders, adjusting the ratio as necessary. The thickness can be estimated using the crystal monitor system by adjusting the density and z-factor, typically ranging between 300 to 500 nm based on the powder ratio. Finally, the last two layers of TiO_2_ and ITO can be deposited using DC sputtering targets to finalize the device.

## Results and discussion

### Layer thickness optimization

**Figure 2:**
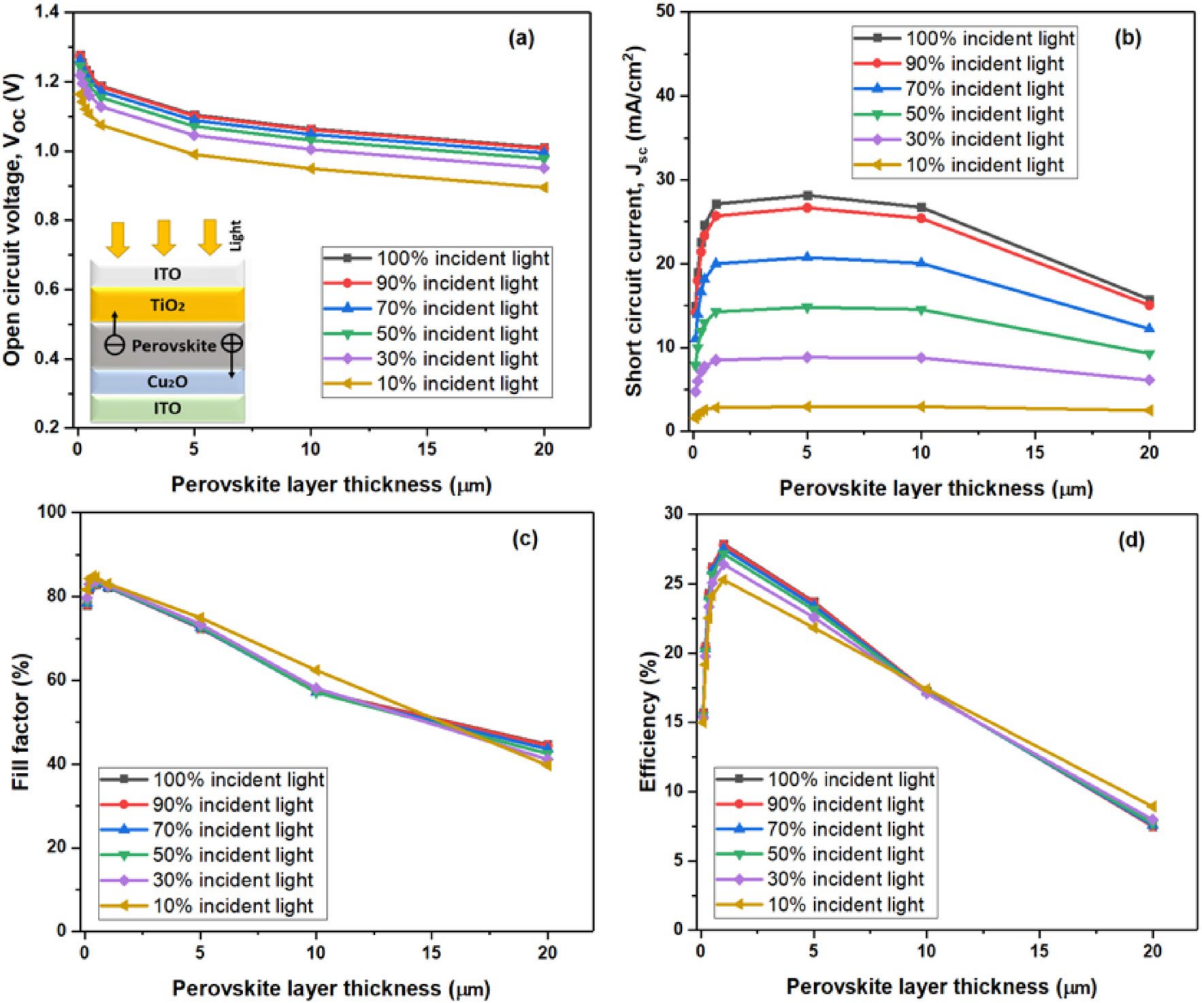
Variation in perovskite solar cells' performance for the incident solar spectrum from the ETL side in the range of 100–20,000 nm of absorber layer thickness. The variables include (**a**) open circuit voltage (V_oc_), (**b**) short circuit current (J_sc_), (**c**) fill factor (FF), and (**d**) efficiency.

**Figure 3:**
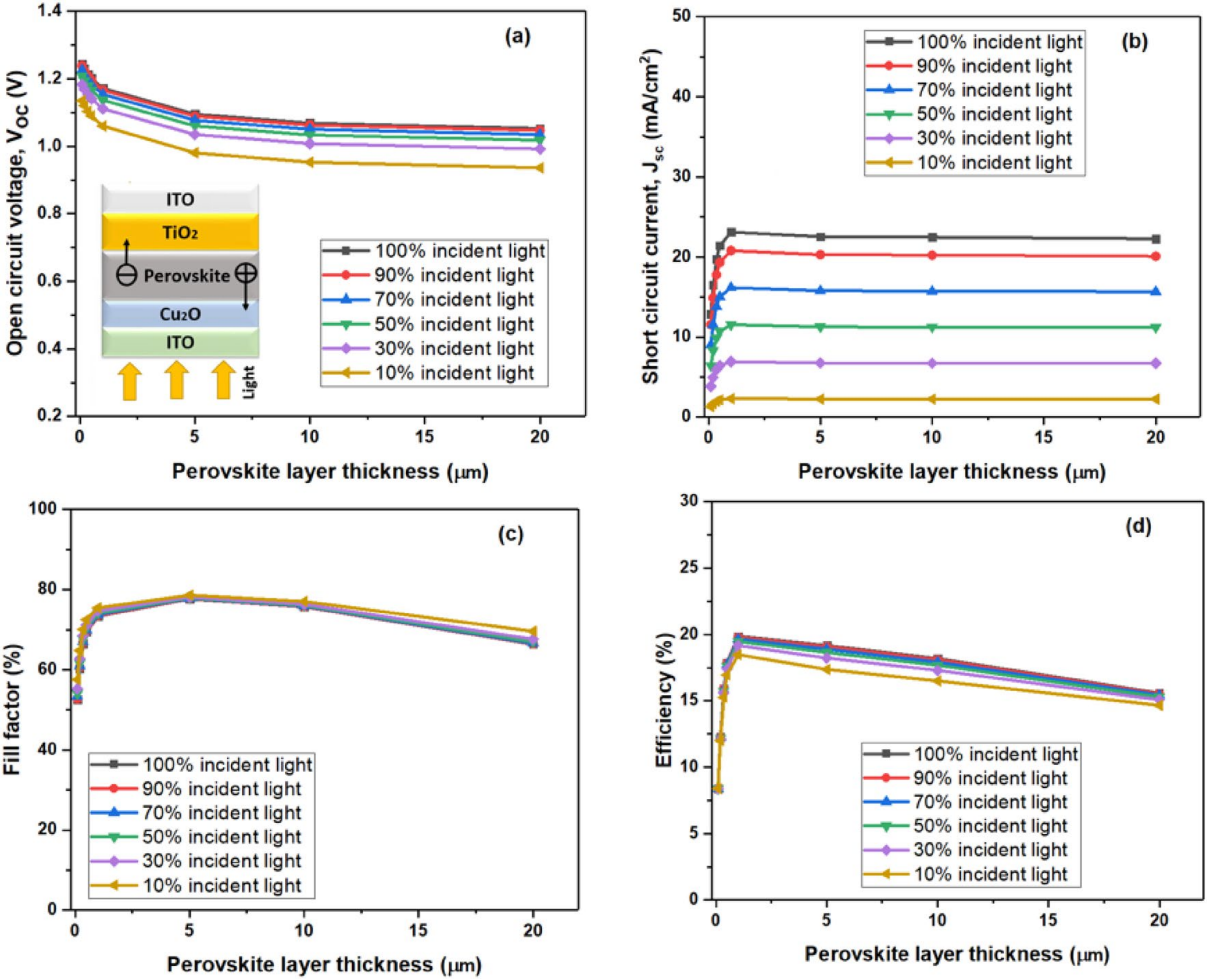
Variation in perovskite solar cells' performance for the incident solar spectrum from the HTL side in the range of 100–2000 nm of absorber layer thickness (**a**) open circuit voltage (Voc), (**b**) short circuit current (Jsc), (**c**) fill factor (FF), and (**d**) efficiency.

This section discusses layer thickness optimization, specifically regarding the electron transport layer (ETL) and hole transport layer (HTL). Based on recommendations from earlier studies, the HTL and ETL layers in the particular case reported in this study were assigned fixed thickness values of 200 nm and 140 nm, respectively^1^. Following the observation of greater power conversion efficiency (PCE) and short-circuit current (J_SC_) with a thickness of 500 nm as opposed to 1000 nm, the latter thickness was selected for a number of reasons. Although the organic/inorganic perovskite system's active layer typically has a thickness of 400 nm, a larger active layer was chosen for this work to improve the device's capacity for light absorption and to optimize charge production. Figures 2 and 3 illustrate the methodical testing that was conducted on the perovskite absorber layer at a range of thicknesses, from 100 nm to 20,000 nm. Beyond this thickness, there's a diminishing return in performance gains due to the saturation of key parameters such as short-circuit current. Additionally, the slight decrease in efficiency observed beyond 1000 nm may be attributed to challenges related to material uniformity and defect management. The study's findings showed that a perovskite absorber thickness of 1000 nm was the ideal operating temperature for the bifacial solar cell. While these optimum thickness values were shown to be beneficial for the situations that this study looked at, it's crucial to remember that these values may not apply to other configurations and that more research is needed to see if they do.

We analyzed the variations in essential parameters that affect solar cell efficiency with respect to the absorber layer thickness, spanning from 100 to 20,000 nm, utilizing SCAPS. The outcomes are depicted in Fig. 2. As expected, the short-circuit current shows an upward trend with an increase in absorber layer thickness across all cell types, peaking at around 1000 nm before reaching a plateau. After this, incident photons are fully absorbed, resulting in a marginal decrease that eventually leads to the maximum generated current (Fig. 2b). The following equation illustrates the extremely low band-to-band recombination rate which is the cause of the slight decrease observed beyond 1000 nm, as defect-assisted carrier recombination was excluded.

$$U\left( {cm^{ - 3} .s^{ - 1} } \right) = B \, \left( {np - n_{i}^{2} } \right)$$

Here, n and p are the carrier densities under excitation, n_i_ is the perovskite's intrinsic density, and B is a constant specific to the material. We used the constant B of GaAs (7.2 × 10^−10^ cm^−3^.s^−1^), which has a direct gap of 1.43eV, since there was no measured value available. But as the thickness exceeds 1000 nm, as Fig. 2d illustrates, there is a noticeable decrease in the power conversion efficiency and open-circuit voltage (Voc), with the fill factor being unchanged. These findings suggest that the performance of PSCs is influenced by the thickness of the perovskite layer. When the perovskite thickness exceeds 300 nm, the Voc experiences a significant decrease attributed to heightened recombination, resulting in insufficient charge carrier separation at the interface of the ETL and absorber layer. Additionally, this inadequate charge carrier separation may stem from elevated series resistances, further contributing to a gradual decline in J_sc_.

On the other side, the TiO_2_ layer serves dual purposes as an electrically conductive layer and a hole-blocking layer, facilitating the collection of electrons from the absorber layer while preventing hole attachment. Given its role as an electrically conductive layer, the thickness of the TiO_2_ layer directly impacts its electrical conductivity. As the TiO_2_ layer thickens, its electrical conductivity diminishes due to increased photon absorption at the ETL layer, consequently heightening charge transfer resistance and ultimately diminishing the PSC's performance.

The decision to maintain fixed thickness values for the HTL and ETL layers was motivated by considerations specific to the bifacial cell design, aiming to explore how variations in the absorber layer thickness, within the established ETL and HTL parameters, affected the solar cell's overall performance. The saturation of both J_sc_ and V_oc_, leading to a subsequent saturation in PCE, becomes apparent beyond a 1000 nm thickness when the value of the recombination constant B is small, whereas it decreases when B has a higher value. Remarkably, the reduction in V_oc_ is more noticeable for greater thicknesses of the perovskite layer in comparison to the short-circuit current. Acknowledging that Voc is dependent on J_sc_ as well as the device's saturation current, J0—which is influenced by the layer's thickness—will help to explain this phenomenon:$V_{oc} = \frac{kT}{e} \, L{\text{n}}\left[ {\frac{{{\text{I}}_{{{\text{sc}}}} }}{{J_{0} }} + 1} \right]$.

**Figure 4:**
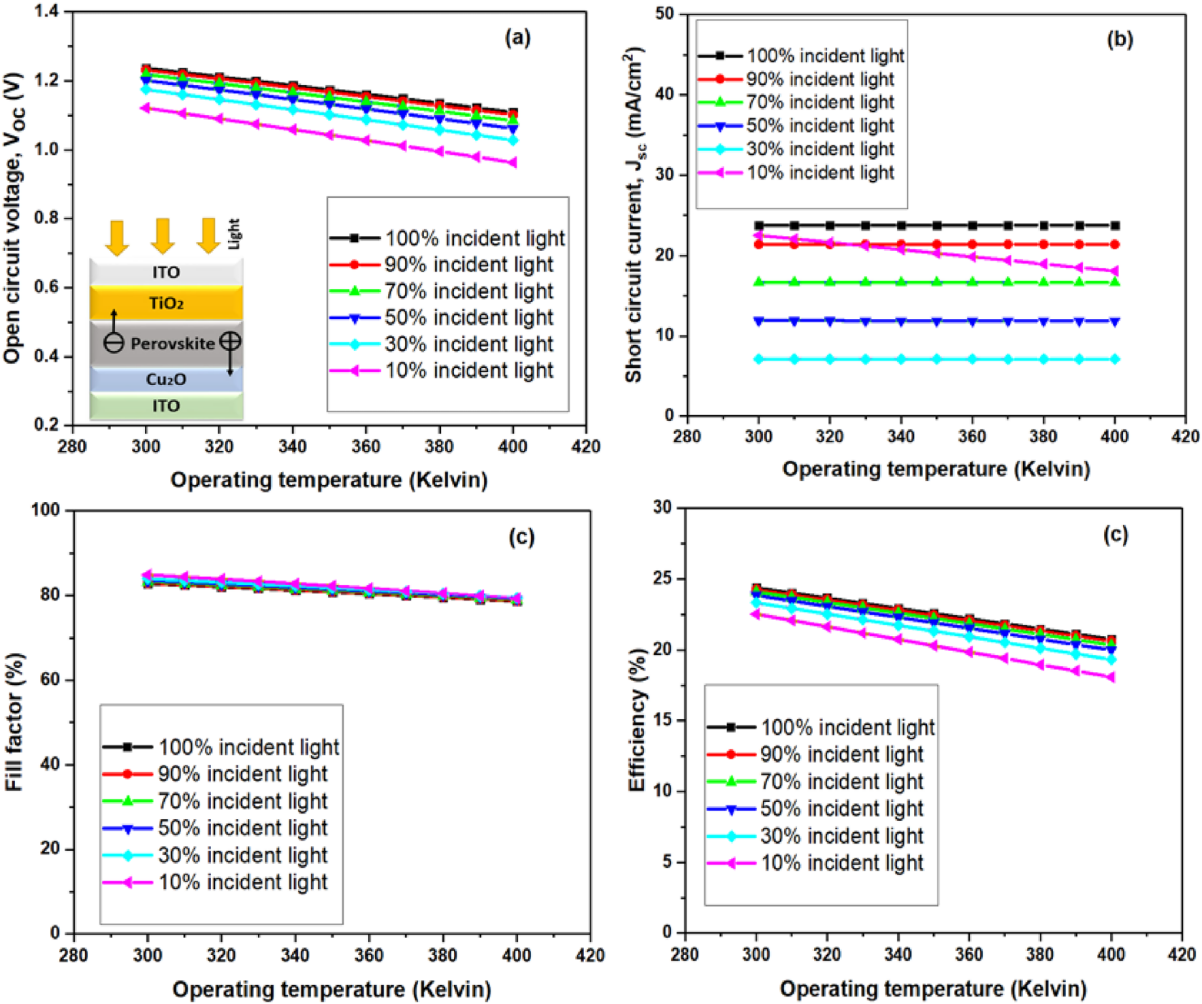
Device performance for the incident solar spectrum from the ETL side at different operating temperatures for the absorber layer thickness of 350 nm (**a**) Open circuit voltage (V_oc_), (**b**) short circuit current (J_sc_), (**c**) fill factor (FF), and (**d**) efficiency of perovskite solar cells.

It is clear that the device's reliance on its saturation current, J_0_, has the greatest impact on the fluctuation of the open-circuit voltage (V_oc_). Moreover, Fig. 4c clearly shows that, in spite of changes in the absorber's thickness, the fill factor (FF) stays comparatively constant. A high short-circuit current and open-circuit voltage could be the primary causes of the bifacial-based device's superior efficiency, as evidenced by the fill factor of the incident light from the ETL side closely resembling that of other devices.

Certainly, the transmittance data is crucial for analyzing the bifaciality of a solar cell. Transmittance data provides insight into how much light passes through the solar cell from both the front and back sides, which is essential for understanding its bifacial performance. In our work, bifaciality refers to the ability to generate electricity from both incident sunlight on its front surface and reflected sunlight on its rear surface. Therefore, we have used the different intensity of incoming solar energy from 10 to 90% to evaluate the efficiency of light transmission through the cell, impacting its overall performance. In our simulation work, the limitation is to incorporate the transmission data for each layer to assess how effectively light penetrates through the solar cell material. This analysis is particularly important for bifacial solar cells, as it allows researchers to optimize the cell's design and materials to maximize light absorption and conversion efficiency from both sides. Furthermore, comparing transmittance data between different cell configurations or materials provides valuable insights into the impact of these factors on bifacial performance. As calculated, variations in the thickness of the solar cell layers affects light transmission, ultimately influencing bifaciality^34,35^. In general, bifacial solar cells are characterized by their bifaciality factor, which reprents the ratio of the nominal efficiency on the rear side to that on the front side. The nominal efficiency is determined by dividing the nominal power (under STC) in [kWp] by the area of the PV module [m^2^].

### Operating temperature's effects on bifacial solar cells

**Figure 5:**
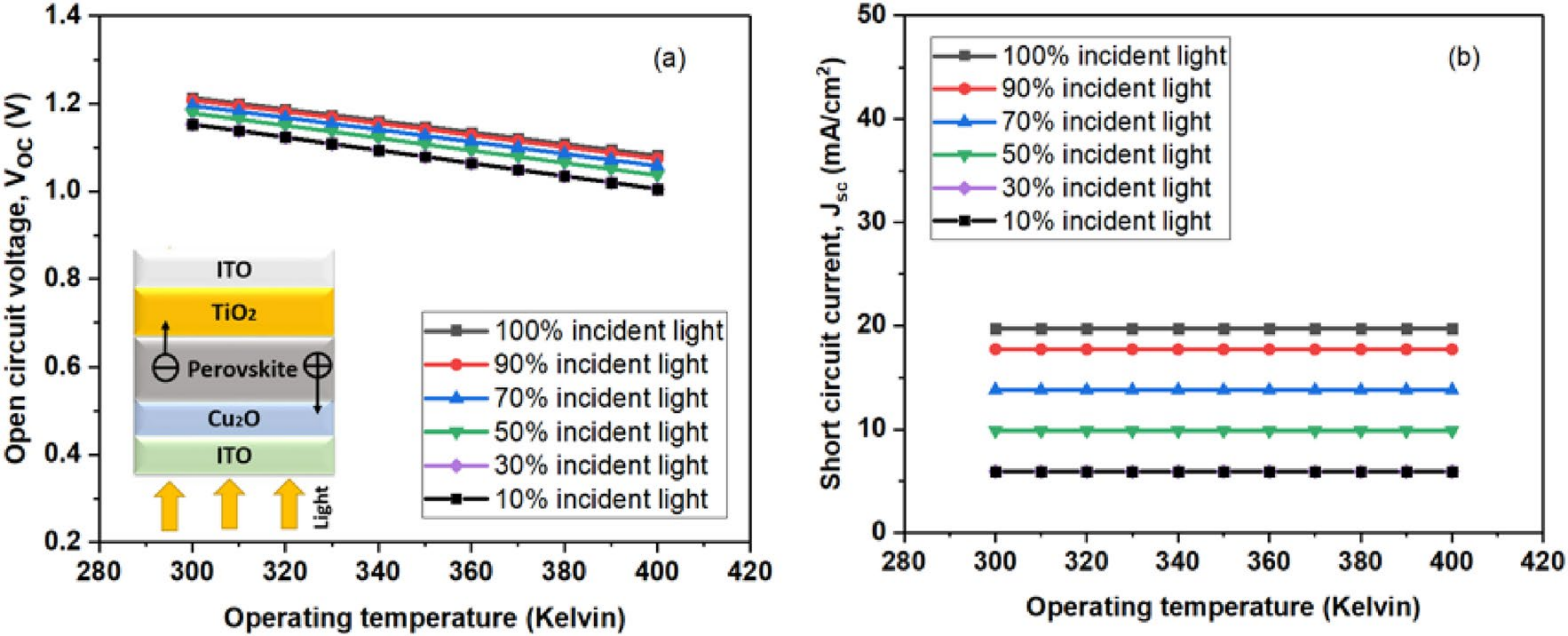
Device performance for the incident solar spectrum from the ETL side at different operating temperatures for the absorber layer thickness of 350 nm (**a**) Open circuit voltage (V_oc_), (**b**) short circuit current (J_sc_).

A parallel investigation was conducted for bifacial perovskite layer while incident light coming from both sides as shown in Figs. 4 and 5, encompassing a temperature range of 300 K to 400 K for variable operation, with aim of understanding its impact on cell performance.

As the operating temperature rises, the efficiency decreases and it makes sense to assume that, in accordance with the analysis results, parameters like electron and hole mobility, carrier concentrations, and band gaps of the materials would change at higher temperatures.

### Doping concentration's effects on TiO_2_ ETL layers

**Figure 6:**
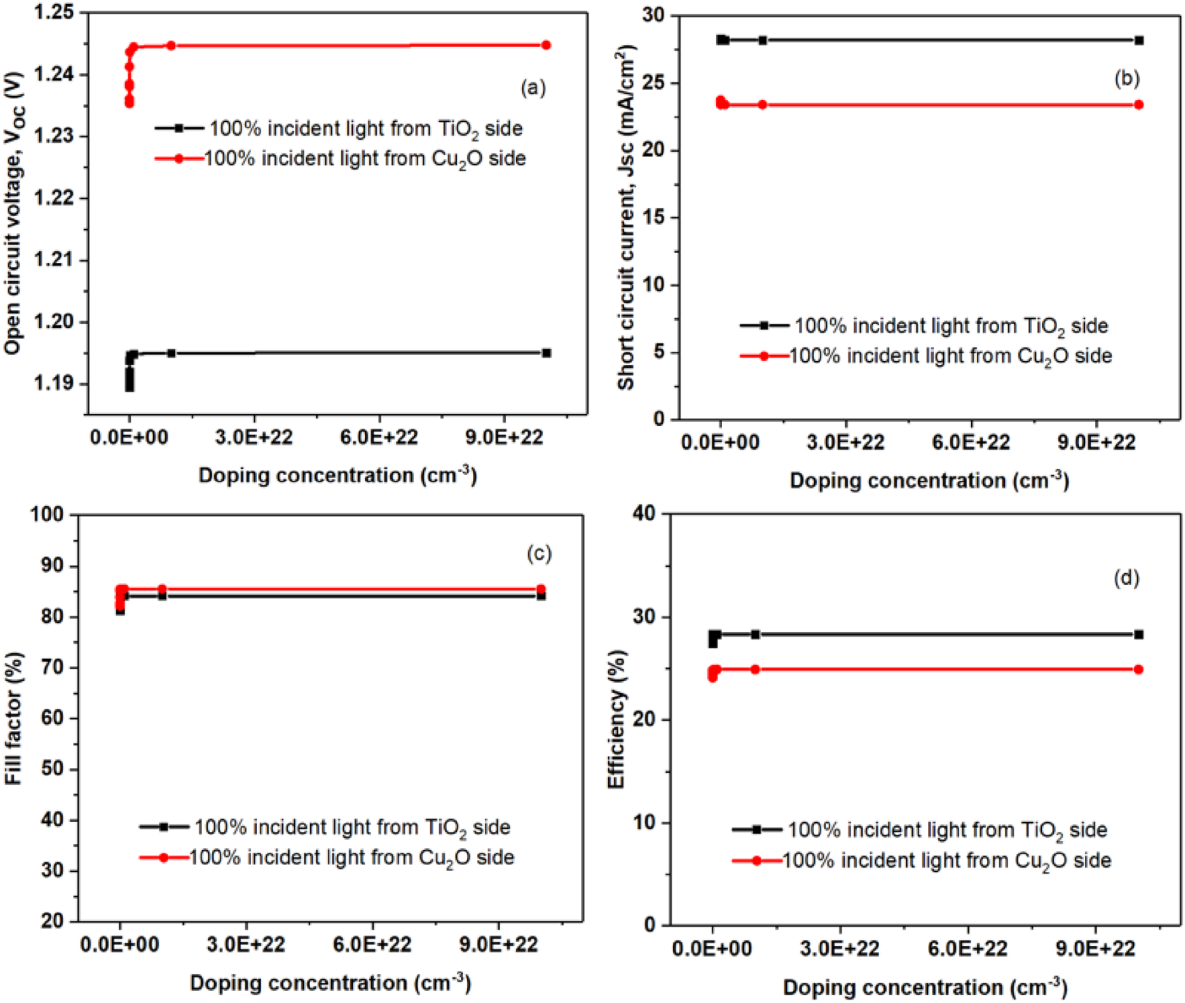
Performance variations in solar cells within the absorber layer thickness range of 350 nm for different TiO2 ETL doping concentrations (**a**) Open circuit voltage (V_oc_), (**b**) Short circuit current (J_sc_), (**c**) Fill factor (FF), and (**d**) Efficiency (η) of perovskite solar cells.

Throughout the computational process, the efficiency of the bifacial solar cell was assessed at different doping concentration level of the ETL from 1.00E + 12 cm^−3^ to 1.00E + 23 cm^−3^ as shown in Fig. 6. Notably, the highest efficiency was consistently observed at the 1000 nm thickness of the perovskite absorber layer. But when the doping concentration rises above 1.00E + 19 cm^−3^, the efficiency saturates. When the light is shone from the HTL side, there is a noticeable drop in both the power conversion efficiency and the open circuit voltage V_oc_, but the fill factor remains approximately constant.

Furthermore, by examining the external quantum efficiency (EQE), we are able to gain a better understanding of the spectrum response of perovskite solar cells. An essential statistic for evaluating the cell's sensitivity to different light wavelengths is EQE. Changes in layer thickness were seen to have observable impacts on light absorption in the perovskite layer, leading to significant changes in EQE throughout the spectrum. Furthermore, it was shown that variations in operating temperature influenced bandgap properties and charge carrier mobility, which in turn affected EQE. Changes in doping concentration—especially in transport layers like TiO_2_—were crucial in controlling carrier transport and recombination rates, which in turn caused notable variations in EQE.

There are limitations of simulations compared to experiments. Our simulations mainly provide insights to predict or optimize device performance, design parameters, or material properties. We can perform parameter sensitivity analysis to understand the impact of various factors on device performance. This includes varying material properties, device geometry, operating conditions, or fabrication parameters to identify key factors influencing performance. In addition, we have demonstrated how simulations can aid in design optimization and cost reduction by minimizing the need for iterative experimental testing.

## Conclusion

Performance variations in solar cells within the absorber layer thickness range of 350 nm for different TiO_2_ ETL doping concentrations. We have demonstrated that the bifacial structure has potential to improve perovskite-based solar cells' efficiency even further. We can synthesis copper oxide (Cu_2_O) with hole mobility up to 250 cm^2^/Vs by using the sputtering technique. The actual performance of the device is anticipated to be significantly influenced by the efficiency of the copper oxide/perovskite interface. The bifacial structure cell may function better if experimental trials that validate the use of copper oxide as a hole-transporting material (HTM) turn out to be successful. Additionally, it might shield the perovskite from moisture, which would strengthen the device's resistance to deterioration. The preliminary numerical analysis using SCAPS-1D confirms that PCE reaches up to 27% for the proposed structure. After shining the cell from p- side there is a gain in efficiency up to 15%, hence, a total conversion efficiency of 30% is achievable. The calculated bifaciality factor (BF) for this structure is 71%. Material parameters that were taken out of pertinent literature were used in computations. Material parameters that were taken out of pertinent literature were used in computations. Therefore, it keeps the door open to develop efficient BPSCs with n-i-p structure. Nevertheless, it is important to remember that our study is mostly theoretical in approach, and more experimental validation is required to verify the effectiveness of our suggested solutions. However, our results offer insightful information for the creation of more reliable and efficient perovskite-based solar cells, which might lead to important breakthroughs in the field of renewable energy technology.

## Data Availability

The data that support the findings of this study are available from the corresponding author, [MIH], upon reasonable request.
